# Durable Disease Control Following Multimodal Therapy in Prostate Cancer With a Massive Pelvic Lymph Node Metastasis

**DOI:** 10.7759/cureus.94012

**Published:** 2025-10-07

**Authors:** Yoshihiro Kawaguchi, Katsuaki Chikui, Etsuyo Ogo, Makoto Nakiri, Tsukasa Igawa

**Affiliations:** 1 Department of Urology, Saiseikai Futsukaichi Hospital, Fukuoka, JPN; 2 Department of Urology, Kurume University School of Medicine, Kurume, JPN; 3 Department of Radiology, Kurume University School of Medicine, Kurume, JPN

**Keywords:** androgen deprivation therapy, bulky lymph node, docetaxel, multimodal therapy, pelvic lymph node metastasis, prostate cancer, radiotherapy

## Abstract

Prostate cancer with a massive pelvic lymph node metastasis is uncommon, and its optimal management remains uncertain. We describe the case of a 69-year-old man with a cT3aN1M0 prostate cancer and a 10 cm (175 cm³) pelvic lymph node metastasis. He received multimodal therapy consisting of androgen deprivation therapy (ADT), seven cycles of docetaxel, and external beam radiotherapy. This approach led to marked regression of the nodal disease and sustained suppression of prostate-specific antigen (PSA) to <0.01 ng/mL for longer than 14 months without additional systemic treatment. Although the therapeutic strategy deviated from current guideline-based recommendations, the case highlights that combining systemic therapy with radiotherapy can achieve durable disease control in selected patients with locally advanced prostate cancer and massive lymph node involvement.

## Introduction

Androgen deprivation therapy (ADT) combined with radiotherapy is generally regarded as the standard treatment for patients with clinically node-positive (cN1) prostate cancer [1-3]. However, the optimal approach for patients presenting with a massive pelvic lymph node metastasis remains uncertain, as such cases are relatively uncommon and are not specifically addressed in current guidelines. Clinically node-positive (cN1) prostate cancer is reported in approximately 5%-10% of newly diagnosed cases in Western countries and 7%-12% in Japan, whereas cases with massive nodal metastases exceeding 10 cm in diameter are extremely rare, with only a few cases reported in the literature. Bulky nodal metastasis is generally associated with poor prognosis, and the absolute size and volume of nodal disease have been correlated with clinical outcomes [4]. Nevertheless, some reports suggest that carefully selected patients may benefit from intensified multimodal therapy [5,6]. Here, we report a case of prostate cancer with a massive pelvic lymph node metastasis that responded favorably to multimodal therapy, resulting in marked tumor shrinkage and durable disease control.

## Case presentation

**Figure 1:**
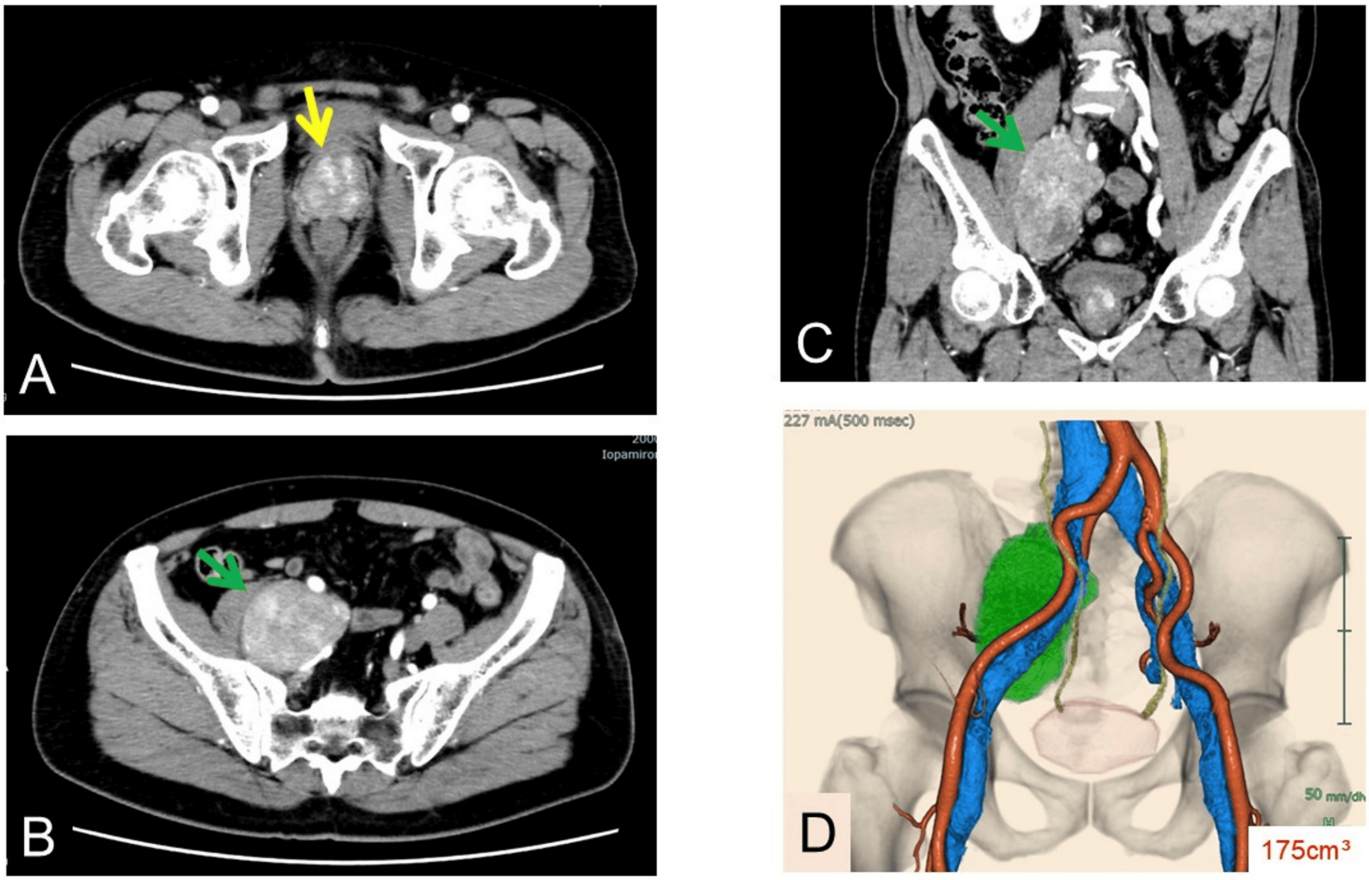
CT images A: Axial contrast-enhanced CT showing the prostate (yellow arrow), measuring approximately 43 × 40 × 39 mm. MRI revealed extracapsular extension on the right lobe, consistent with clinical stage T3a disease. B and C: Axial and coronal CT views showing a right pelvic lymph node metastasis measuring 100 × 62 × 50 mm (green arrows indicate the lymph node). D: Three-dimensional reconstruction using SYNAPSE VINCENT software showing the lymph node volume (green), calculated to be 175 cm³. CT: computed tomography, MRI: magnetic resonance imaging

A 69-year-old man presented with a one-month history of intermittent discomfort and numbness in his right thigh. Initial evaluation at a local clinic included an ultrasound, which raised suspicion of an iliac artery aneurysm and a possible association with the prostate. Due to these findings, he was referred to our hospital for further evaluation. Laboratory tests revealed a markedly elevated prostate-specific antigen (PSA) level of 1,091.7 ng/mL (reference range: <4 ng/mL), while serum lactate dehydrogenase (LDH), alkaline phosphatase (ALP), and urinalysis were within normal limits. Contrast-enhanced computed tomography (CT) demonstrated a markedly enlarged right internal iliac lymph node measuring 100 × 62 × 50 mm, with a calculated volume of 175 cm³ using a 3D analysis system (SYNAPSE VINCENT, Fujifilm, Tokyo, Japan) (Figure 1).

**Figure 2:**
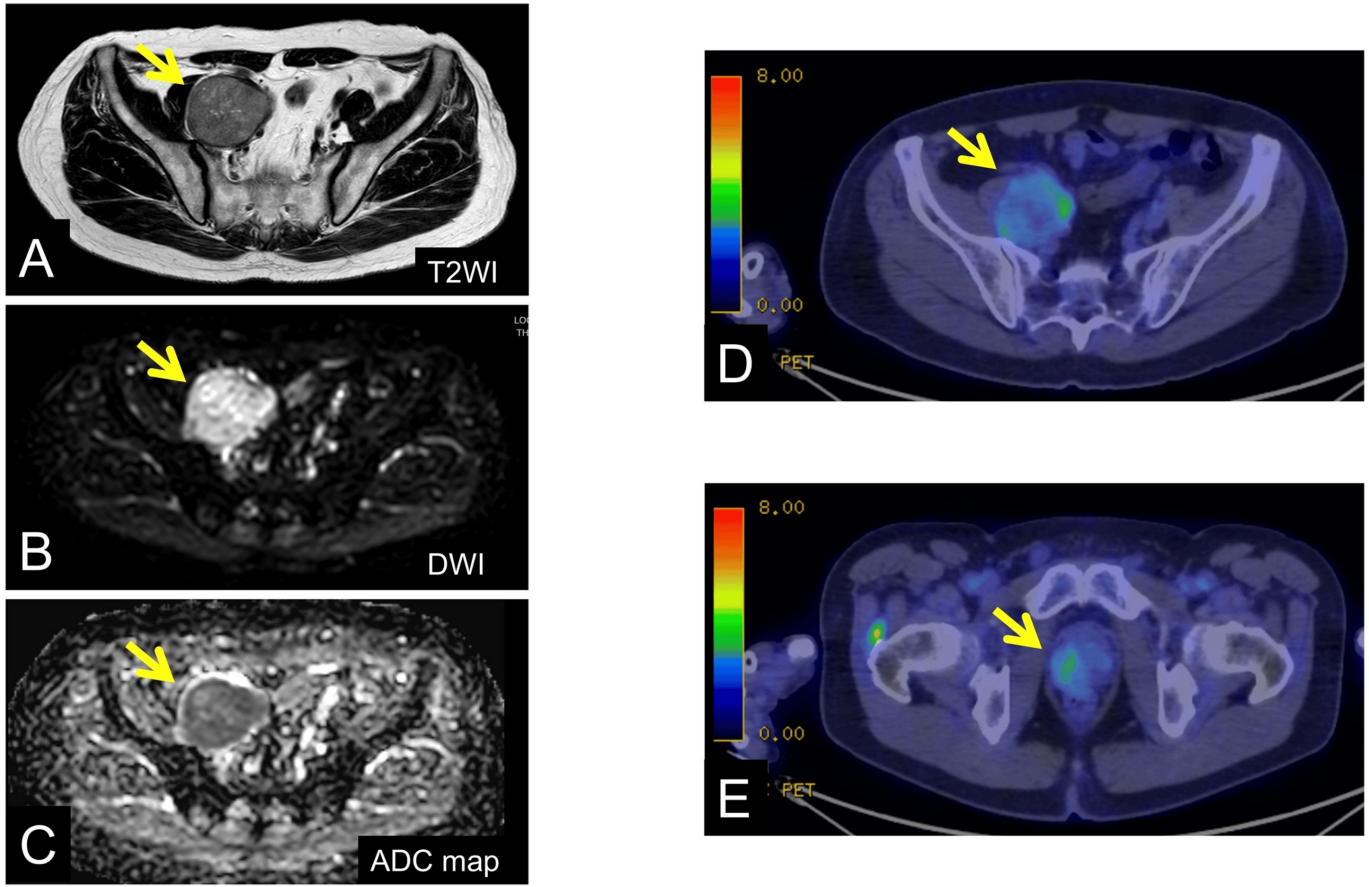
MRI findings and FDG PET-CT images A-C: MRI findings: The yellow arrow indicates the right pelvic lymph node. The lymph node shows no communication with adjacent vessels on T2WI, high signal intensity on DWI, and low signal intensity on the ADC map. D and E: FDG PET-CT images: The yellow arrow indicates (D) the right pelvic lymph node and (E) the right peripheral zone of the prostate, showing increased uptake (SUVmax: 4.4 early/4.7 delayed in the lymph node; 3.5 early/4.2 delayed in the prostate). MRI: magnetic resonance imaging, FDG: fludeoxyglucose, PET-CT: positron emission tomography-computed tomography, T2WI: T2-weighted imaging, DWI: diffusion-weighted imaging, ADC: apparent diffusion coefficient, SUVmax: maximum standardized uptake value

Magnetic resonance imaging (MRI) showed extracapsular extension of the prostate with high signal intensity on diffusion-weighted imaging (DWI) and low signal on the apparent diffusion coefficient (ADC) map (Figure 2).

**Figure 3:**
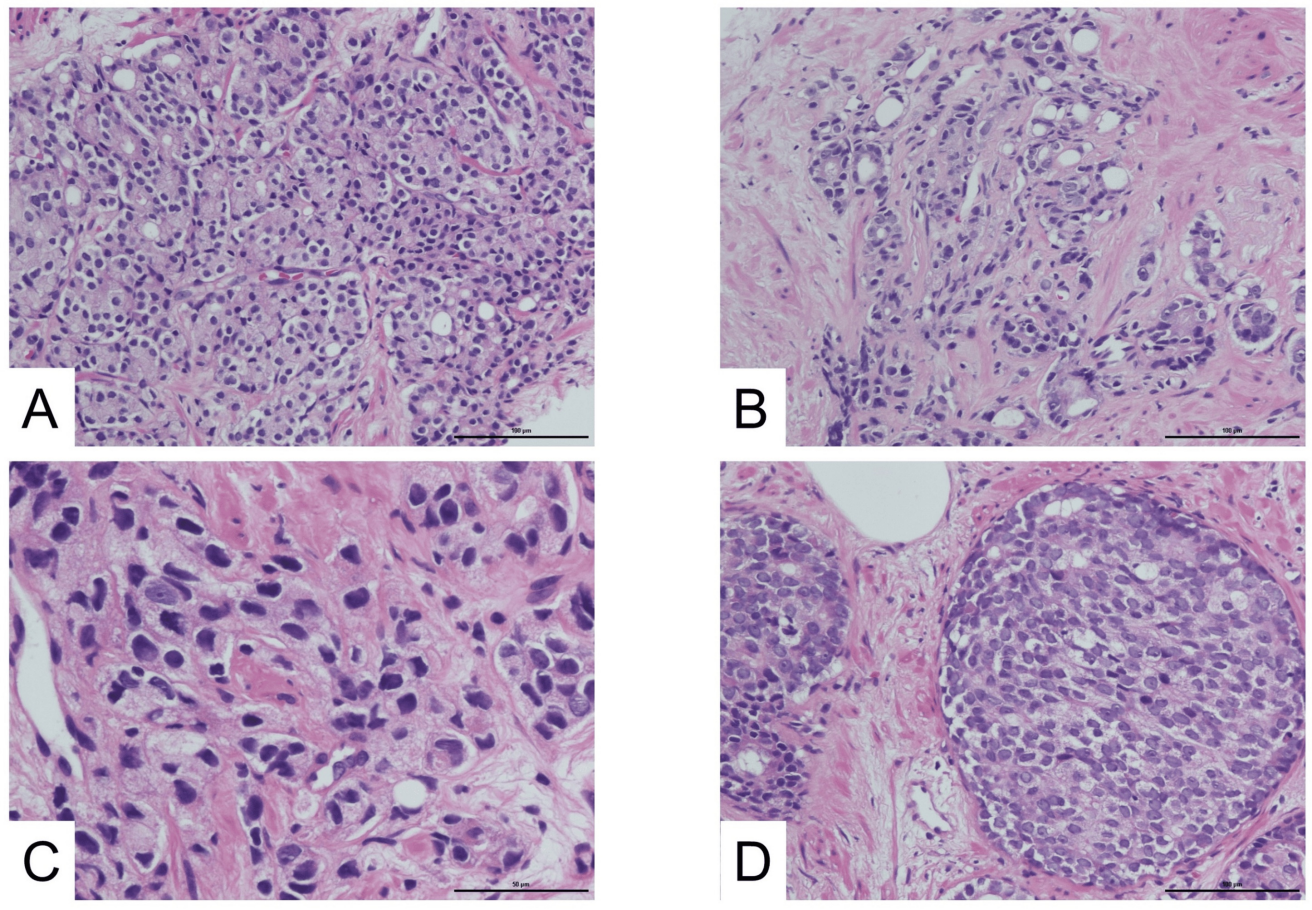
Histopathological findings of the prostate biopsy A and B: Low-magnification views (scale bar = 100 μm) showing fused and poorly formed glands, respectively. C and D: High-magnification views (scale bar = 50 μm) showing single-cell infiltrates with cords and solid growth patterns, consistent with high-grade prostate adenocarcinoma.

Positron emission tomography-CT (PET-CT) demonstrated increased uptake in the right peripheral zone of the prostate (maximum standardized uptake value (SUVmax): 3.5 (early phase) → 4.2 (delayed phase)) and in the enlarged lymph node (SUVmax: 4.4 → 4.7). Bone scintigraphy revealed no evidence of osseous metastasis. Histopathological examination of prostate biopsy specimens revealed fused and poorly formed glands with cribriform, solid, and cord-like growth patterns (Figure 3).

Adenocarcinoma of the prostate was diagnosed, clinical stage cT3aN1M0 with a Gleason score of 4 + 5 = 9, and carcinoma was established in all 12 biopsy cores. ADT was initiated with an LH-RH agonist plus bicalutamide 80 mg/day, which led to an initial decline in PSA. Although radiotherapy was recommended, the patient initially declined. Approximately one year after starting ADT, the PSA began to show six consecutive increases with a doubling time of about 2.5 months. During this period, the patient remained on ADT, and serum neuron-specific enolase (NSE) was mildly elevated, raising suspicion of possible neuroendocrine differentiation and treatment resistance. Based on PSA progression and NSE elevation, docetaxel chemotherapy was initiated two years after the initial diagnosis. Seven cycles were administered (first cycle: 75 mg/m², subsequent cycles: 70 mg/m²), and the patient tolerated treatment well, achieving a marked PSA decline without significant adverse events. At this stage, bicalutamide was discontinued, but ADT with an LH-RH agonist was continued. Around the third year, enzalutamide was introduced but discontinued shortly thereafter due to fatigue at the patient’s request. In the same year, external beam radiotherapy was administered (70 Gy in 28 fractions to the prostate and 50.4 Gy in 28 fractions to the pelvic lymph node).

**Figure 4:**
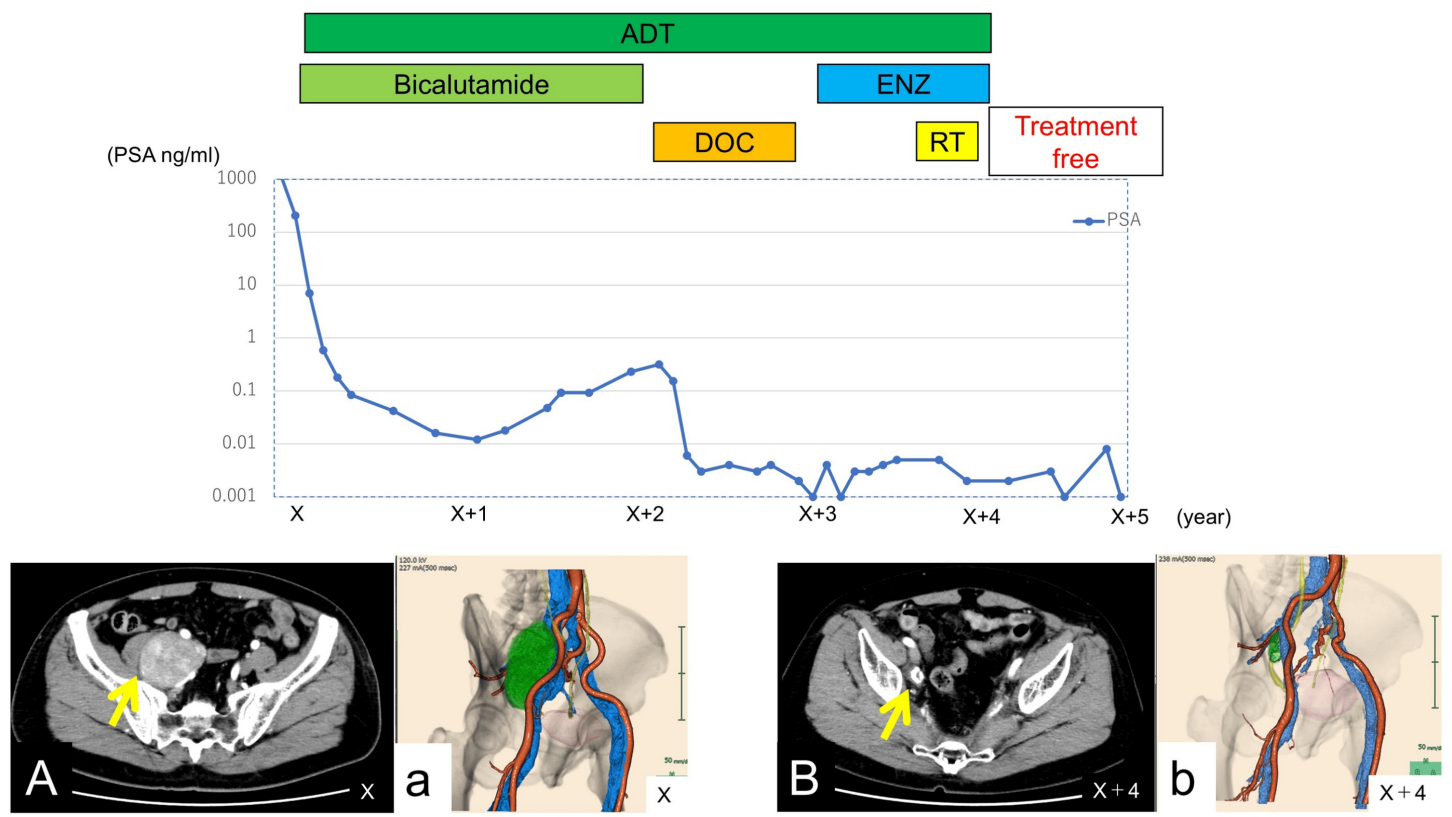
Clinical course of the patient The graph shows the five-year clinical course from the initial diagnosis. X represents the year of the initial diagnosis; X+1, X+2, etc. indicate subsequent years after diagnosis. PSA levels are presented on a logarithmic scale. PSA decreased significantly following multimodal therapy and has remained below 0.01 ng/mL for the past 14 months. Representative CT images are shown to illustrate the radiologic response: panels A and a (yellow arrow, right pelvic lymph node metastasis) at diagnosis, and panels B and b (yellow arrow, residual shrunken lymph node) at four years after treatment (volume reduction from 175 cm³ to 2.7 cm³, 98.5% decrease). PSA: prostate-specific antigen, CT: computed tomography, ADT: androgen deprivation therapy, ENZ: enzalutamide, DOC: docetaxel, RT: radiotherapy

Following multimodal therapy, the lymph node volume decreased from 175 cm³ to 2.7 cm³, representing a 98.5% reduction (Figure 4).

Although further treatment was discontinued at the patient’s own request four years after the initiation of therapy, he has maintained a near-complete response for over 14 months since discontinuation, with PSA persistently <0.01 ng/mL and no evidence of new lesions on follow-up imaging.

## Discussion

This case illustrates that multimodal therapy incorporating ADT, docetaxel, and radiotherapy can achieve durable disease control in prostate cancer with massive pelvic lymph node involvement. Large cohort studies have shown that nodal size and volume correlate with oncologic outcomes in prostate cancer [4]. Therefore, bulky disease is generally considered to portend a poor prognosis. In our patient, however, the favorable course appeared to be more strongly influenced by the treatment strategy (intensive multimodal therapy) than by nodal size alone. Nevertheless, this observation should be interpreted cautiously and cannot be generalized, as unique tumor biology (such as high sensitivity to ADT or radiosensitivity) may also have contributed.

For patients with clinically node-positive (cN1M0) prostate cancer, current guidelines, including those by the National Comprehensive Cancer Network (NCCN) and the European Association of Urology (EAU), recommend long-term ADT combined with external beam radiotherapy as standard therapy [1-3]. In the present case, initial management deviated from guidelines due to patient preference, and docetaxel was introduced later. While trials such as CHAARTED and STAMPEDE demonstrated the benefit of early docetaxel in metastatic hormone-sensitive prostate cancer, its application to non-metastatic node-positive disease is less clear [5,6]. Nonetheless, in this high-risk setting, docetaxel likely contributed to disease control.

The elevation of NSE was another factor influencing clinical decision-making. NSE is a biomarker of neuroendocrine differentiation in prostate cancer and has been associated with poor prognosis and resistance to androgen receptor (AR)-targeted therapy [7]. In our case, although a repeat biopsy was not performed, NSE elevation was regarded as a clinical warning sign of potential neuroendocrine differentiation and prompted earlier introduction of systemic chemotherapy.

The addition of radiotherapy seemed critical in achieving a durable response. Both the prostate and lymph node lesions showed uptake on PET-CT, suggesting that local control was necessary alongside systemic therapy. Previous case reports also describe favorable outcomes with multimodal approaches in patients with bulky nodal disease, although such cases are rare [8,9].

This report has limitations. It describes a single case, and the therapeutic sequence was influenced by patient preference rather than guidelines, limiting generalizability. Moreover, no histological confirmation of neuroendocrine differentiation was obtained. Despite these limitations, this case highlights that multimodal therapy can be a feasible and potentially effective approach for selected patients with massive pelvic nodal metastasis.

## Conclusions

This case highlights that even in prostate cancer, exceptionally large pelvic lymph node metastases may respond favorably to an intensive multimodal treatment approach, including ADT, docetaxel, and radiotherapy. While long-term outcomes remain uncertain and this report represents a single case, the observed durable remission suggests that individualized, aggressive therapy may offer clinical benefit in carefully selected patients with advanced nodal disease. Further studies are needed to establish optimal management strategies for such rare presentations.
